# Effects of Ion-Transporting Proteins on the Digestive System Under Hypoxia

**DOI:** 10.3389/fphys.2022.870243

**Published:** 2022-09-14

**Authors:** Yiwei Xiang, Dongdong Fan, Qimin An, Ting Zhang, Xianli Wu, Jianhong Ding, Xiaolin Xu, Gengyu Yue, Siqi Tang, Qian Du, Jingyu Xu, Rui Xie

**Affiliations:** ^1^ Department of Gastroenterology, Digestive Disease Hospital, Affiliated Hospital of Zunyi Medical University, Zunyi, China; ^2^ Collaborative Innovation Center of Tissue Damage Repair and Regeneration Medicine, Zunyi, China

**Keywords:** hypoxia, ion, ion-transporting protein, digestive, system

## Abstract

Hypoxia refers to a state of oxygen limitation, which mainly mediates pathological processes in the human body and participates in the regulation of normal physiological processes. In the hypoxic environment, the main regulator of human body homeostasis is the hypoxia-inducible factor family (HIF). HIF can regulate the expression of many hypoxia-induced genes and then participate in various physiological and pathological processes of the human body. Ion-transporting proteins are extremely important types of proteins. Ion-transporting proteins are distributed on cell membranes or organelles and strictly control the inflow or outflow of ions in cells or organelles. Changes in ions in cells are often closely related to extensive physiological and pathological processes in the human body. Numerous studies have confirmed that hypoxia and its regulatory factors can regulate the transcription and expression of ion-transporting protein-related genes. Under hypoxic stress, the regulation and interaction of ion-transporting proteins by hypoxia often leads to diseases of various human systems and even tumors. Using ion-transporting proteins and hypoxia as targets to explore the mechanism of digestive system diseases and targeted therapy is expected to become a new breakthrough point.

## 1 Introduction

Oxygen is vital to the human body. The human body allows oxygen to enter the body through lung ventilation, and the oxygen is exchanged in the alveoli and delivered to the capillaries through the alveolar-capillary exchange system. In capillaries, oxygen combines with hemoglobin to enter cells of various tissues, and finally participates in energy production in mitochondria in cells to meet the metabolic needs of cells (Ortiz-Prado et al., 2019). However, each organ in the human body has different metabolic levels and functional states, so under physiological conditions, most organs are in their unique physiological normoxic state (Nakayama and Kataoka, 2019). For example, the partial pressure of oxygen (PO_2_) in the brain is 30–48 mmHg, and the PO_2_ in the superficial epidermis of the kidney is 72 ± 20 mmHg. The PO_2_ in the liver is 55.5 ± 21.3 mmHg, the PO_2_ around the portal vein is about 60–65 mmHg, while in the perivenous area it drops to about 30–35 mmHg (Kietzmann, 2019). There is a unique oxygen gradient in the human gut. The partial pressure of oxygen in the colon wall is 42–71 mmHg, the PO_2_ near the recess-cavity interface is 5–10 mmHg, and the PO_2_ in the ascending colon cavity and sigmoid colon cavity are 11 and 3 mmHg, respectively. The PO_2_ of the small intestinal wall is 59 mmHg, the tip of the villi is about 22 mmHg, and the PO_2_ of the small intestinal lumen is < 10 mmHg (Singhal and Shah, 2020). The PO_2_ of the pancreas is about 40 mmHg (Lo et al., 2013; Ortiz-Prado et al., 2019). However, in the event of conditions including respiratory failure, insufficient blood flow to end organs, dysfunctional or low levels of hemoglobin, or chemically induced hypoxia, the partial pressure of oxygen will fall below physiological normoxic levels. The oxygen will be limited, and the cells will be in a hypoxic environment. A hypoxic microenvironment can trigger changes in cellular metabolism and trigger different molecular responses. The hypoxia response can be part of the normal physiological activities of the human body, but it mainly mediates the pathological process and promotes the progression of the disease.

### 1.1 Hypoxia and its Regulating Factors

**FIGURE 1:**
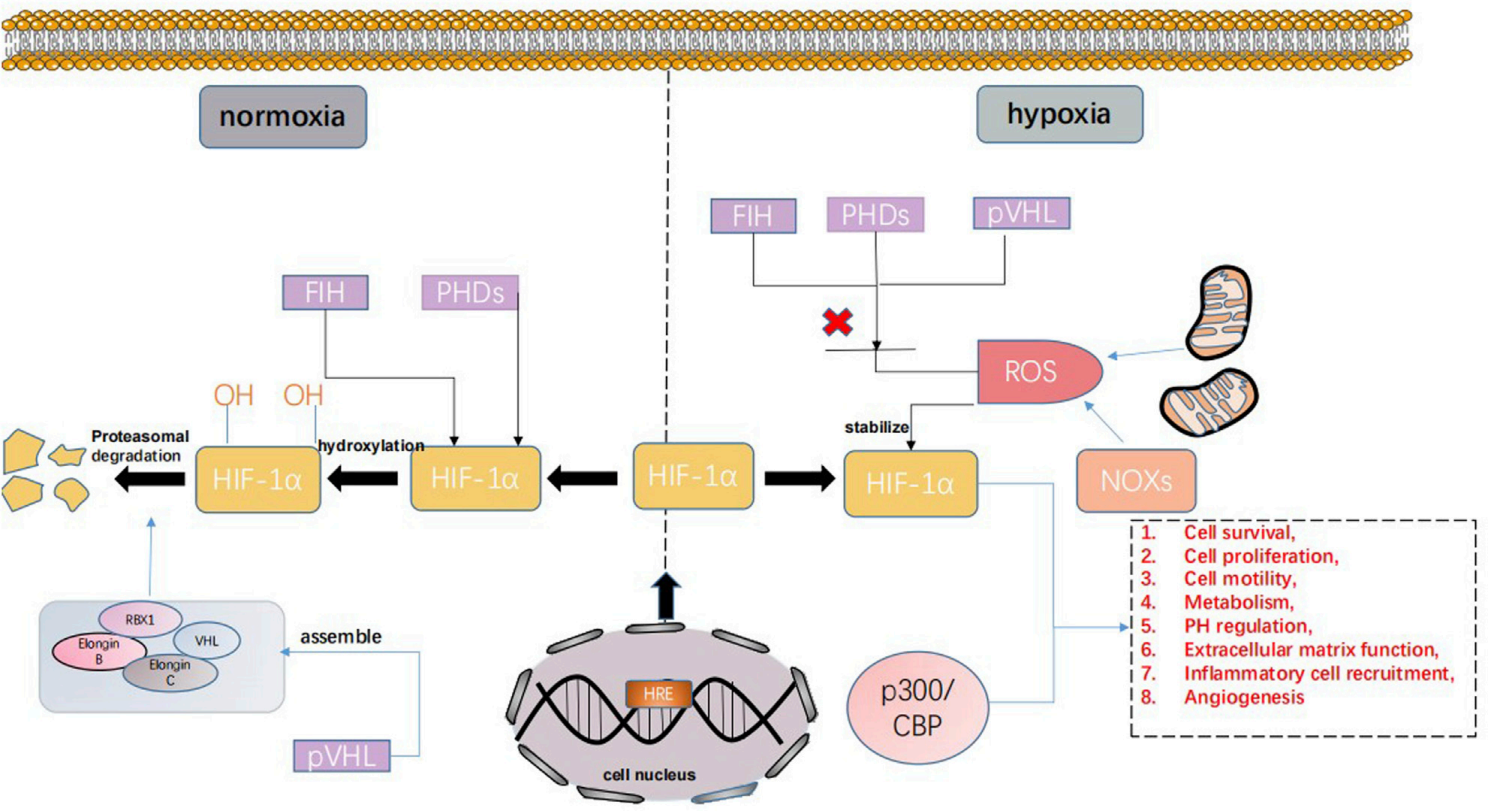
HIF can be expressed in different ways under normoxia and hypoxia. Under normoxic conditions, the hypoxia-inducible factor-1α subunit can bind the von Hippel-Lindau tumor suppressor gene product (PVHL). pVHL mediates the ubiquitin–proteasome, which is composed of RBX1, Elongin B, Elongin C, and VHL. The ubiquitin–proteasome pathway rapidly degrades HIF. In contrast, under hypoxic conditions, the hypoxia-inducible factor-1α subunit becomes stable and interacts with coactivators, such as p300/CBP, and the transcriptional activity of hypoxia-inducible factor-1α is enhanced. Under hypoxic conditions, it has become the main regulator of many hypoxia-induced genes involved in cell survival, proliferation, exercise, metabolism, pH regulation, extracellular matrix function, inflammatory cell recruitment, angiogenesis, etc.

The main regulator of human tissue homeostasis under hypoxia is the hypoxia-inducible factor (HIF) family. Hypoxia-inducible factor was originally discovered as an enhancer of the erythropoietin (EPO) gene in the process of identifying hypoxia response elements (HREs) (Ke and Costa, 2006). The HIF family has three main members, namely, HIF-1, HIF-2, and HIF-3. The functional subunit forms of these three members are HIF-1α, HIF-2α, and HIF-3α. Currently, the effect of HIF-3α is unclear. Studies have only found that HIF-3α can inhibit the transcription of HIF-1/2α and act as a dominant negative regulator of HIF-1/2α activity. HIF-2α has a similar structure to HIF-1α, but its expression pattern is different because in contrast to widespread HIF-1α, HIF-2α is expressed only in certain tissues. Therefore, HIF-1α is most widely known (Loboda et al., 2010; Duan, 2016). The stability and activity of HIF-1α are regulated by posttranslational modification, hydroxylation, acetylation, and phosphorylation. Hypoxia-inducible factor-1α can interact with various protein factors, including PHD, pVHL, ARD-1, p300/CBP, RBX1, Elongin B, and Elongin C. Under normoxic conditions, the hypoxia-inducible factor-1α subunit can be triggered by the hydroxylation of proline and the acetylation of lysine in a polypeptide segment called the oxygen-dependent degradation (ODD) domain, and combined with von Hippel-Lindau tumor suppressor gene product (pVHL), pVHL mediates the ubiquitin–proteasome composed of RBX1, Elongin B, Elongin C, and VHL and rapidly degrades HIF through the ubiquitin–proteasome pathway. In contrast, under hypoxic conditions, the hypoxia-inducible factor-1α subunit becomes stable and interacts with coactivators, such as p300/CBP. Thus, the transcriptional activity of hypoxia-inducible factor-1α is enhanced, and under hypoxic conditions, it becomes the main regulator of many hypoxia-inducible genes (Lee et al., 2004). These genes are involved in cell survival, proliferation, movement, metabolism, pH regulation, extracellular matrix function, inflammatory cell recruitment, and angiogenesis (Joseph et al., 2018). HIF-1 regulates these genes and leads to various pathological processes (Figure 1). In conclusion, HIF is an important factor regulating the oxygen balance in the human body and an important target of hypoxia-mediated activities (Li et al., 2020).

### 1.2 The Role of Hypoxia and its Regulating Factors in the Physiological and Pathological Processes of the Digestive System

The impact of hypoxia and its regulatory factors on the human body has always been a hot topic and studies have increasingly shown that hypoxia can play an important role in the human body (Fuentes and Christianson, 2016). In the past, a large number of studies on the effects of hypoxia on the human body tended to focus on the cardiovascular, respiratory, urinary, and nervous systems, such as myocardial apoptosis and hypertrophy (Eigel et al., 2004; Chu et al., 2012), pulmonary artery contraction and remodeling (Dong et al., 2012; Simonneau et al., 2014; Parpaite et al., 2016), nerve cell apoptosis (Aarts et al., 2003), and renal ischemia reperfusion (Kita et al., 2007; Liu et al., 2018). However, the effects of hypoxia on the human body are not limited to the cardiovascular, respiratory, nervous, urinary, and other systems. In recent years, the effects of hypoxia on the digestive system have also received increasing attention. Increasing evidence shows that hypoxia and its regulatory factors can also be widely expressed in various organs of the digestive system and play an extremely important role in the physiological and pathological processes of the digestive system.

#### 1.2.1 The Physiological Role of Hypoxia in the Digestive System

Hypoxia can be used as a physiological signal that can affect many physiological processes in the digestive system (Hubbi and Semenza, 2015). For example, as an important digestive organ, the liver performs various functions necessary for maintaining systemic homeostasis. The liver is the central metabolic organ responsible for maintaining blood glucose levels, ammonia metabolism, endogenous metabolic by-products of biotransformation and metabolism of xenobiotics, and bile synthesis. All of these processes require many pathways and enzymatic reactions to run in parallel in the most efficient manner. To achieve this, the liver parenchyma exhibits a functional organization called metabolic zonation. It is currently recognized that both Wnt/β-catenin pathway and Hedgehog (Hh) signaling play a decisive role in metabolic partitioning (Benhamouche et al., 2006; Matz-Soja et al., 2013). In hypoxic environment, hif-1a and HIF-β promote the expression of Wnt/β-catenin target genes including Dkk-4, Lef-1, and Tcf-1. And HIF-1α can directly bind to the promoters of Lef1 and TCF1 genes, thereby greatly enhancing the transcriptional activity of β-catenin (Mazumdar et al., 2010). At the same time, hypoxia can induce the expression of Hh ligand SHh and the pathway activity marker Patched1, thereby inducing a systemic Hh response (Bijlsma et al., 2009). Through these two pathways, hypoxia and its regulators promote the formation of hepatic metabolic zonation (Kietzmann, 2017). Induced hepatic stem cells (iHepSCs) are lineage-reprogrammed cells derived from murine embryonic fibroblasts with self-renewal and bipotential differentiation properties, which hold great potential as hepatocyte therapy donors. Physiological hypoxia can accelerate the G1/S transition through the p53-p21 signaling pathway, thereby enhancing stemness characteristics and promoting the proliferative capacity of iHepSCs. In addition, short-term hypoxia preconditioning enhances the hepatic differentiation efficiency of iHepSCs, and long-term hypoxia promotes cholangiocyte differentiation but inhibits hepatic differentiation of iHepSCs (Zhi et al., 2018). Studies have shown that physiological hypoxia can assist the formation of the barrier function of the gastrointestinal tract. Tight junctions are the backbone that form the upper intestinal barrier, and claudins are integral membrane proteins in the intestinal barrier responsible for the selective permeability of tight junctions (Ivanov et al., 2010). The hypoxic intestinal environment induces the production of HIF-1β, which maintains CLDN1 expression by binding to the hypoxia-responsive element sequence in the gene promoter (Saeedi et al., 2015). Xenobiotic clearance is an important function of the intestinal epithelial barrier. P-glycoprotein, also known as multidrug resistance protein 1, has broad substrate specificity. In the process of xenobiotic clearance, P-glycoprotein is the main effector of xenobiotic transport into the lumen. P-glycoprotein is transcriptionally regulated by HIF-1 (Comerford et al., 2002; Zheng et al., 2015).

#### 1.2.2 The Pathological Role of Hypoxia in the Digestive System

As a common environmental stress factor, hypoxia is more involved in the pathological process of the digestive system. First, in the liver, hypoxia-induced overexpression of HIF-2α can inhibit fatty acid β-oxidation, thereby activating peroxisome proliferators to activate the receptor PPARα, which, in turn, induces fat formation in the liver and exacerbates nonalcoholic fatty liver disease (NAFLD) (J. Chen et al., 2019). Hypoxia is a common phenomenon in hepatocellular carcinoma (HCC). Hypoxia stabilizes the transcription factor hypoxia-inducible factor (HIF), and the expression of HIF is closely related to the metastasis of liver cancer. The metastasis of liver cancer requires the support of metastasis-promoting genes, and hypoxia/hypoxia-inducible factor-1α has been shown to be a central regulator of many metastasis-promoting genes that can activate metastasis-promoting genes; thus, it ultimately participates in every step of liver cancer metastasis (Wong et al., 2014). In the pancreas, pancreatic ductal adenocarcinoma (PDAC) is characterized by fewer blood vessels, strong invasiveness, and a very poor prognosis. PDAC has strong invasion and migration ability because under hypoxic conditions, pancreatic cancer-associated fibroblasts (CAFs) are stimulated by hypoxia to produce insulin-like growth Factor 1 (IGF1), and IGF1/IGF1R signaling can stimulate the invasion and migration activity of PDAC cells (Hirakawa et al., 2016). In the esophagus, hypoxia can promote the growth and metastasis of esophageal squamous cell carcinoma (ESCC). Under hypoxic conditions, hypoxia stimulates the production of HIF-1α, and HIF-1α can induce the expression of microRNA (miRNA), a posttranscriptional regulatory factor. MicroRNAs can stimulate the growth and metastasis of esophageal squamous cell carcinoma (ESCC) (Zhang et al., 2019). In the stomach, a key factor underlying gastric cancer invasion and metastasis is the epithelial-mesenchymal transition (EMT). Hypoxia-inducible factor-1α not only is an independent prognostic factor of gastric cancer but also, under hypoxic conditions, can stimulate the overexpression of prostate cancer gene expression marker 1 (PCGEM1). The overexpression of prostate cancer gene expression marker 1 (PCGEM1) can affect the epithelial-mesenchymal transition (EMT), thereby promoting the invasion and metastasis of gastric cancer cells (Zhang J et al., 2019). In the intestine, the severity of inflammatory bowel disease was found to be positively correlated with the expression of HIF-1a (Cummins and Crean, 2017). In colorectal cancer (CRC), the Hippo signaling pathway is a central pathway that regulates intestinal growth. Related protein 1 (YAP1) is a downstream effector of the Hippo signaling pathway. YAP1 is an important regulator of proliferation, organ size, and cell differentiation. HIF-2α can directly increase the activity of cancer cells by upregulating YAP1 activity to promote the growth of colorectal cancer cells (Ma et al., 2017).

Overall, numerous studies have shown that hypoxia can play an important role in the physiological processes and diseases of the digestive system. However, to date, how hypoxia specifically affects the digestive system, especially in some diseases and tumors, its mechanism, and its function have not been fully elucidated. A deeper understanding of the mechanism of hypoxia and its regulatory factors in the digestive system could help us further understand the development, changes, and prognosis of the disease and discover new drug treatment targets.

### 1.3 Correlation Between Ion-Transporting Proteins and Hypoxia

In recent years, through the exploration of the mechanism of hypoxia and its regulatory factors, it has been discovered that hypoxia can affect various human systems by regulating the expression of ion-transporting proteins and interacting with various ion-transporting proteins. First, for human cells, ion-transporting proteins constitute an extremely important class of proteins that are distributed on the cell membrane and form pores in the cell membrane, strictly controlling the inflow or outflow of ions in the cell or organelle (Keynes, 1975). Ions are important cell signals in the human body. Changes in ions in cells are often closely related to extensive physiological and pathological processes in the human body. In addition, there is amazing molecular diversity in ion-transporting proteins. Ion transporters comprise a wide range of ion channels, exchangers, pumps, and ionotropic receptors. They are based on ion selectivity (sodium channel, potassium channel, chloride channel, calcium channel, proton channel, and nonselective channel), gating mechanism (voltage gating, ligand gating, cyclic nucleotide gating, light gate, and mechanically sensitive), or localization (plasma membrane and intracellular) for classification (Venter et al., 2001), Kv, Kca, Ki, NCX, and TRP channel families (Venkatachalam and Montell, 2007), STIM, NHE, etc. Because of their diversity, limited tissue distribution, and important role in regulating key cell functions, they are very attractive therapeutic or diagnostic targets in many diseases (Kaczorowski et al., 2008).

**FIGURE 2:**
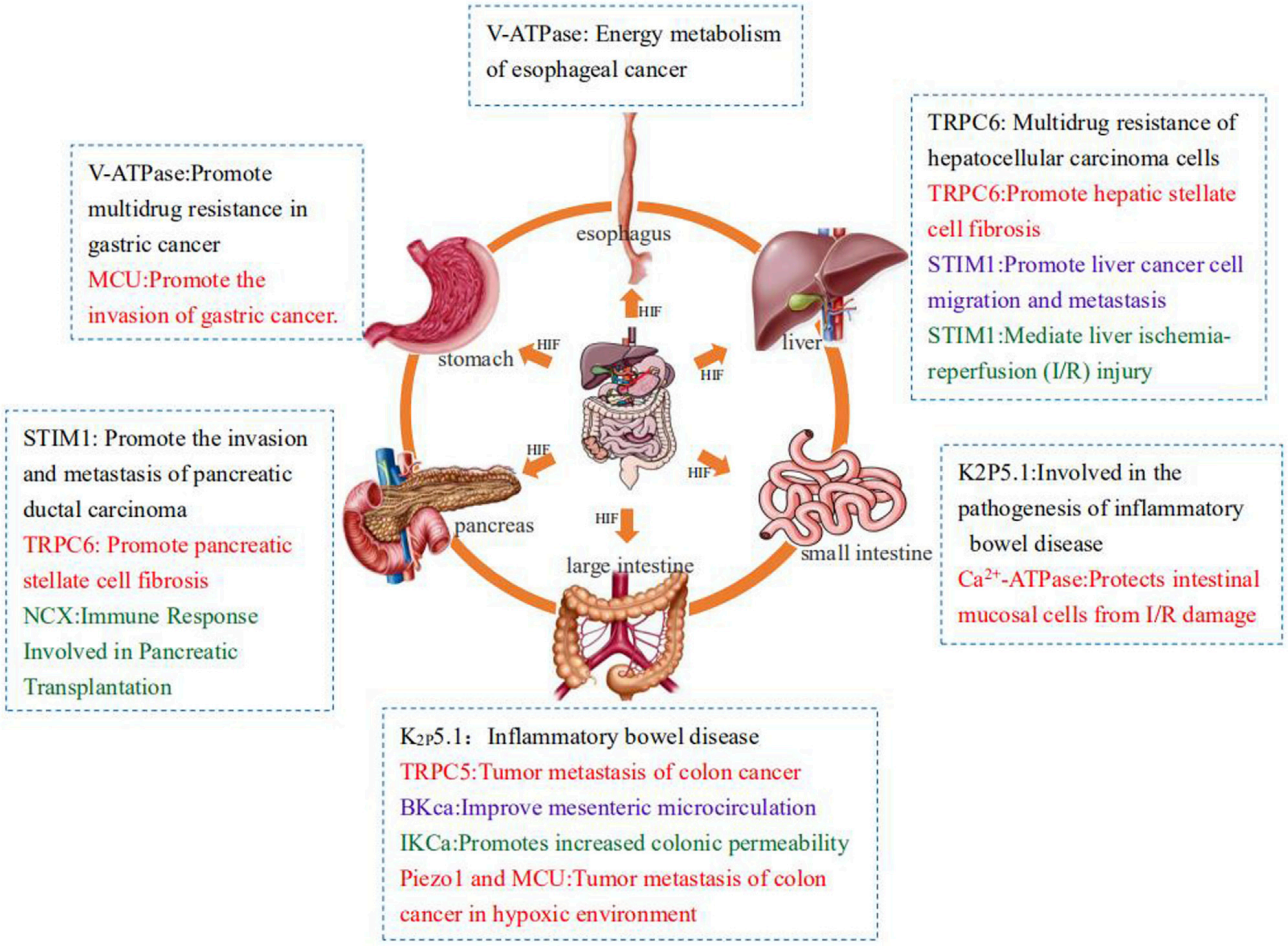
Summary of the effects of ion transporters on the digestive system under hypoxia.

However, numerous studies have proven that under normal circumstances, hypoxia and its regulatory factor HIF-1 can regulate the transcription and expression of ion-transporting protein-related genes such that the related ion-transporting proteins are overexpressed or inhibited. Sometimes, activated ion-transporting proteins will in turn affect the expression of HIF-1, causing it to activate or silence. When the various systems of the human body are under hypoxic stress, the regulation and interaction of hypoxia on ion-transporting proteins often leads to the occurrence of related diseases and even tumors. This article aims to review the effects of ion-transporting proteins on the digestive system under hypoxia to provide new ideas for clinical treatment (Figure 2).

## 2 The Role of Ion-Transporting Proteins in the Digestive System Under Hypoxia

### 2.1 The Effect of Vacuolar H^+^-ATPase on Energy Metabolism of Esophageal Carcinoma Cells Under Hypoxia

#### 2.1.1 Physiological Role of V-ATPase and Energy Metabolism of Esophageal Cells

The vacuolar (H^+^)-ATPase (V-ATPase) is an ATP-dependent proton pump. V-ATPase is composed of V0 and V1 domains. The V0 domain consists of five distinct subunits involved in proton transport (Forgac, 2007). On the other hand, the V1 domain consists of 8 different subunits responsible for the hydrolysis of ATP (Forgac, 2007). V-ATPase is mainly responsible for the maintenance of PH in human plasma and cells, the transport and secretion of intracellular and intracellular membranes, the processing and degradation of some macromolecules, and the coupled transport of some small molecules such as neurotransmitters and ATP (Cipriano et al., 2008). Like cells in other parts of the body, under normal circumstances, esophageal cells usually use glycolysis to convert glucose in the cytoplasm into pyruvate. Pyruvate is further metabolized in mitochondria through the tricarboxylic acid (TCA) cycle and the electron transport chain (ETC) through oxidative phosphorylation (OxPhos) to generate energy storage adenosine triphosphate (ATP) (Pelicano et al., 2006).

But we were surprised to find that V-ATPase plays a completely different role in esophageal cancer cells, and the energy metabolism of esophageal cancer cells is also completely different from the tricarboxylic acid cycle of normal cells.

#### 2.1.2 V-ATPase can Positively Regulate HIF-1, Thereby Enhancing the Expression of Key Genes of Glycolysis and Promoting Glycolysis in Esophageal Cancer Cells

Esophageal cancer is one of the deadliest malignant tumors, and the prognosis is often poor (Nassri et al., 2018). Although there have been advances in chemotherapy and radiotherapy in recent years, due to its high mortality rate, the 5-year survival rate of patients with esophageal squamous cell carcinoma (ESCC) is very low (up to 30%–45%) (Russo and Franceschi, 1996). One of the main reasons is that, in contrast to normal cells, tumor cells heavily rely on glycolysis to produce energy, even with sufficient oxygen levels, which allows them to maintain a high proliferation rate and resist apoptosis signals (Liu et al., 2001).

Studies have shown that in the process of glycolysis, the key genes of glycolysis, glucose transporter 1 (Glut1), hexokinase II (HK_2_), and lactate dehydrogenase A (LDHA) activation are often dependent on HIF-1 (Nagao et al., 2019). In esophageal cancer cells, vacuolar H^+^-adenosyl triphosphate (V-ATPase) is highly expressed. V-ATPase is particularly important for maintaining the pH environment required for tumor growth. At the same time, V-ATPase can promote the expression of mammalian target of rapamycin (MTOR) (Buller et al., 2011). MTOR increases glucose uptake and expression of glucose transporter 1 (GLUT_1_). In addition, experiments showed that V-ATPase can also promote HIF-1 and other glycolytic genes such as HK_2_, phosphofructokinase-1 (PFK_1_), enolase 1 (ENO_1_), PKM_2_, LDHA, and pyruvate dehydrogenase thiamine kinase isoenzyme (PDK_1_) expression (Son et al., 2019). Therefore, in the process of glycolysis of esophageal cancer cells, V-ATPase itself can activate the transport of glucose and positively regulate HIF-1, so as to enhance the expression of key genes of glycolysis, promote glycolysis, and provide energy for the proliferation, growth, and metastasis of esophageal cancer cells. For the treatment of esophageal cancer, how to inhibit its glycolytic pathway seems to provide a new idea for the development of new targeted anticancer drugs, and HIF-1 and V-ATPase may also be potential targets for inhibiting glycolysis (Son et al., 2016).

### 2.2 The Regulatory Role of Ion Transporters in Gastric Diseases Under Hypoxia

#### 2.2.1 Under Hypoxia, Vacuolar H^+^-ATPase can Promote the Multidrug Resistance of Gastric Cancer Cells

##### 2.2.1.1 The Emergence of Multidrug Resistance in Gastric Cancer

Gastric cancer is the second leading cause of death worldwide (Ferro et al., 2014). Sixty-five percent of gastric cancer patients are often in the advanced or metastatic stage at the time of diagnosis (Hundahl et al., 2000). The combination of chemotherapy and targeted therapy provides hope of survival for patients with advanced gastric cancer. However, tumor cells sometimes develop resistance to multiple cytotoxic drugs that reduces the effectiveness of chemotherapy, known as multidrug resistance (Ozben, 2006). The main mechanisms of MDR production include reducing drug accumulation in tumor cells, altering intracellular drug distribution, increasing detoxification, reducing drug-target interactions, increasing DNA repair, altering cell cycle regulation, and uncoupling pathways that link cell damage to apoptosis (Martínez-Lacaci et al., 2007; Gillet and Gottesman, 2010). The most dominant form of resistance to chemotherapy has been correlated with two MDR transporters including P-glycoprotein (P-gp), multidrug resistant protein1 (MRP_1_) (Szakács et al., 2006). They can often be overexpressed in malignant cells and can pump a variety of anticancer drugs out of the cell, resulting in lower levels of the intracellular drugs needed for effective treatment (Rees et al., 2009). Therefore, it is necessary to explore the regulatory pathways of these transporters.

##### 2.2.1.2 Vacuolar H^+^-ATPase Increases HIF-1α Translation to Promote P-Gp and MRP1 Expression

As we described above, the main physiological role of V-ATPases in the human body is to maintain the homeostasis of cellular pH. But in gastric cancer cells, we surprisingly found that the activity of V-ATPase was greatly increased. Activated V-ATPase can promote the expression of mTOR. mTOR, a master regulator of protein synthesis and cell growth, increases the translation of hypoxia-inducible factor 1α (HIF-1α) (Advani, 2010). In the hypoxic environment formed by gastric cancer cells, hypoxia-inducible factor-1α (HIF-1α) can also regulate the transcription of a large number of hypoxia-related genes including multidrug resistance genes (Semenza, 2002; Lin et al., 2014). Among them, the expression of HIF-1α can induce a significant increase in the translation of P-gp and MRP_1_ (L. Liu et al., 2008), thereby reducing intracellular drug accumulation and enhancing the drug resistance of esophageal cancer cells. Therefore, both V-ATPase and HIF-1α provide effective therapeutic targets, and reducing the expression of V-ATPase and HIF-1α can increase the effectiveness of chemotherapeutic drugs and even reverse the drug resistance of gastric cancer cells (Chen et al., 2012).

#### 2.2.2 Mitochondrial Calcium Uniporter Regulates Migration, Invasion, Angiogenesis, and Growth of Gastric Cancer Cells Under Hypoxic Conditions

The mitochondrial calcium uniporter (MCU) is a highly selective calcium channel and the primary pathway for calcium entry into mitochondria. In the hypoxic microenvironment of gastric cancer tissue, MCU is highly expressed. The overexpressed MCU can significantly increase the mitochondrial membrane potential level, and after the mitochondrial membrane potential level is increased, the invasive ability of gastric cancer cells is significantly improved. MCU may promote the proliferation of gastric cancer cells by increasing the level of mitochondrial membrane potential. The extracellular matrix (ECM) plays an important role in the invasion and metastasis of gastric cancer. Overexpression of MCU can cause calcium disturbance in gastric cancer cells. Matrix metalloproteinases are zinc- and calcium-dependent proteases that degrade ECM components, promote angiogenesis, and regulate cell adhesion. MCU can regulate matrix metalloproteinases to disrupt ECM homeostasis and promote the invasion and metastasis of gastric cancer cells (Gan et al., 2018). MCU overexpression also significantly promotes the expression of HIF-1α and vimentin, and suppresses the expression of E-cadherin in gastric cancer cells, while HIF-1α can induce the proliferation, migration, and invasion of gastric cancer cells by promoting the expression of VEGF, suggesting that MCU may regulate the expression of VEGF through HIF-1α. Inhibition of E-cadherin promotes epithelial-mesenchymal transition (EMT). EMT is the process by which cells transition from a proliferative epithelial phenotype to a migratory and invasive mesenchymal phenotype. MCU can promote the migration and invasion of gastric cancer cells by regulating the EMT process *in vitro* and *in vivo*. Therefore, targeting MCU may inhibit the migration, invasion, angiogenesis, and growth of gastric cancer cells, providing a new idea for the treatment of gastric cancer (Wang et al., 2020).

### 2.3 The Regulatory Role of Ion Transporters in Intestinal Diseases Under Hypoxia

#### 2.3.1 Hypoxia and K_2_P Channels are Involved in the Pathogenesis of Inflammatory Bowel Disease

##### 2.3.1.1 K_2_P5.1 Channel is Up-Regulated in Inflammatory Bowel Disease

Studies have shown that dysregulation of transcription, translation, and posttranslational expression of K^+^ channels is related to the pathogenesis of immune and inflammatory diseases (Ohya et al., 2016). Among them, K_2_P5.1 channel may be involved in the pathogenesis of inflammatory bowel disease. The alkaline pH-activated K^+^ channel (K_2_P5.1) belongs to the two-pore domain K^+^ (K_2_P) channel superfamily. K_2_P5.1 is not only involved in the resting potential of human cells, but also has various physiological functions such as maintaining cell volume and regulating renal bicarbonate reabsorption (Cid et al., 2013; López-Cayuqueo et al., 2015; Williams et al., 2013). But of most interest is its role in autoimmune and inflammatory diseases such as inflammatory bowel disease. In the study of autoimmune disease models such as inflammatory bowel disease (IBD), it was found that the expression and activity of K_2_P5.1 were significantly upregulated in the CD^4+^ T cells of the model. However, the mechanism of K_2_P5.1 upregulation remains unclear (Nakakura et al., 2015).

##### 2.3.1.2 Under Hypoxia, HIF can Promote the Occurrence of Inflammatory Bowel Disease by Activating K_2_P5.1

Hypoxia-inducible factor-1α is strongly expressed in T cells infiltrating the inflammatory mucosa in patients with IBD (Higashiyama et al., 2012). Hypoxia-inducible factor-1α plays an important role in the pathogenesis of inflammatory diseases by promoting the expression of inflammatory genes (Taylor and Colgan, 2017). Brazier et al. found that HIF-1 dimers can bind target HIF-1 response element (HRE) regions to activate target gene transcription (Brazier et al., 2005) and clone the common binding site of the K_2_P5.1 promoter and Ets (E-26)-like 1 (ELK-1), whose activity is sensitive to oxygen and is necessary for K2P5.1 transcription initiation. The expression and activity of K_2_P5.1 increase under hypoxia (1.5% O2), and treatment with the HIF inhibitor FM19G11 instead of the selective HIF-2 inhibitor has the opposite effect. This finding indicates that under hypoxia, HIF can promote the occurrence of IBD by activating K_2_P5.1 (Endo et al., 2019).

#### 2.3.2 Under Hypoxia, Transient Receptor Potential Channel 5 (TRPC5) can Promote Colon Cancer Tumor Metastasis Through the HIF-1α-Twist Signaling Pathway

Overexpression of TRPC5 in colon cancer cells promotes cancer cell migration and proliferation by inducing epithelial-mesenchymal transition (EMT). TRPC5 belongs to the TRP (Transient receptor potential) superfamily and is one of the major Ca^2+^ regulatory channels in the intestine. Epithelial-mesenchymal transition (EMT) is closely related to the invasive metastasis of tumor cells (Thiery et al., 2009). Several studies have shown that the occurrence of EMT is associated with calcium influx in colon cancer cells. In colon cancer, overexpressed TRPC5 leads to massive Ca^2+^ influx, which reduces the expression of E-cadherin. On the other hand, the expression of interstitial biomarkers N- cadherin and vimentin is significantly increased. Loss of function of the adhesion junction protein E-cadherin is regarded as a major hallmark and fundamental event of EMT (Kalluri and Weinberg, 2009). Further studies have revealed the molecular mechanism of how TRPC5 affects E-cadherin of EMT through Ca^2+^ influx. The major transcription factors that bind to the E-cadherin promoter and directly repress its transcription are the ZeB, Snail, and Twist families (Lee and Nelson, 2012; Perez-Moreno et al., 2001). The transcription factor Twist is highly expressed in colonic cells. The expression of Twist is regulated by HIF-1α, which can regulate the expression of Twist by directly binding to the hypoxia-responsive element (HRE) in the proximal promoter of Twist in the hypoxic environment of colon cancer cells (Yang et al., 2008). At the same time, HIF-1α itself is also a calcium-sensing factor, and the overexpression of TRPC5 can promote the increased translation of HIF-1α. Therefore, overexpression of TRPC5 promotes the migration and proliferation of colon cancer cells through the TRPC5/HIF-1α/Twist signaling pathway, and TRPC5 and HIF-1α may become potential therapeutic targets for colon cancer (Chen et al., 2017).

#### 2.3.3 Piezo1 and MCU are Involved in Colon Cancer Metastasis in Hypoxic Environment

Colon cancer is the leading cause of cancer-related deaths worldwide and, like other tumors, is characterized by migratory, invasive, and metastatic capabilities. Tumor cell mobility is affected by multiple signaling cascades, including multiple ion channels and transporters (Schwab and Stock, 2014). Piezo1, also known as FAM38A, is a member of the PIEZO family, which includes Piezo1 and Piezo2. Piezo1 protein is a component of mechanically activated cation channels. It directly senses mechanical forces and translates environmental signals into intracellular Ca responses, and is widely expressed in a variety of cells and tissues, including tumor cells (Miyamoto et al., 2014; Liu Q et al., 2018). MCU is an evolutionarily conserved Ca^2+^ channel that plays a role in intracellular Ca^2+^ signaling in mitochondria (Ren et al., 2017). HIF-1α, a Ca^2+^ sensitive factor, has been shown to be involved in tumor cell metastasis. In the hypoxic environment of colon cancer, highly expressed Piezo1 activates the MCU. MCU can regulate the concentration of intracellular calcium ions to promote the expression of HIF-1α. VEGF is involved in the migration, invasion, and metastasis of tumor cells. VEGF has been identified as a downstream target activated by HIF-1. Therefore Piezo1 and MCU are likely to play a role in colon cancer cell metastasis through the Piezo1-MCU-HIF-1α-VEGF pathway (Sun et al., 2020).

#### 2.3.4 Under Acute Hypoxia, BKca Channel can Promote Mesenteric Vasodilation, Thereby Improving Mesenteric Microcirculation

Studies have shown that acute hypoxia affects the electrophysiological properties of guinea pig mesenteric arterial smooth muscle cells. BKCa channels belong to a heterogeneous family of Ca^2+^-activated K^+^ channels. Like most cells *in vivo*, K^+^ channel is the main ion channel in the cytoplasmic membrane of smooth muscle and it contributes significantly to resting membrane potential. Activation of the channel can lead to K^+^ cell efflux and membrane hyperpolarization (Guntur et al., 2021). Acute hypoxia can activate the outward current mediated by the BKca channel in the guinea pig mesenteric artery, thereby significantly enhancing the outward current. The activity and current magnitude of this channel directly affect vascular hyperpolarization and vasodilation. When the mesenteric artery smooth muscle cells are acutely hypoxic, the BKca channel on the membrane is activated to cause K^+^ efflux. Therefore, vascular smooth muscle cells are hyperpolarized to relax the blood vessels and improve mesenteric microcirculation (Ma et al., 2011).

#### 2.3.5 IKCa Activation Promotes Increased Colonic Permeability Under Chemical Hypoxia

Chemical hypoxia can increase colonic permeability. Studies have shown that intestinal permeability may be related to basolateral membrane K^+^ channel activity. Metabolic stress secondary to chemical hypoxia causes a rapid increase in the activity of Ca^2+^-sensitive intermediate conductance K^+^ (IK Ca) channels in the basolateral membrane of natural human intestinal epithelial cells. It can greatly increase the whole cell conductance on the basal side of human colonic crypts and double the permeability of colonic membrane cells. Increased mucosal permeability may lead to bacterial translocation, sepsis, and multiple organ failure. The increase in colon permeability caused by chemical hypoxia can be prevented by IKCa channel inhibition, which may be a new way to prevent the harmful effects of increased intestinal permeability (Loganathan et al., 2011).

#### 2.3.6 Hypoxic Preconditioning Increases Ca^2+^-ATPase Activity and Protects Intestinal Mucosal Cells From I/R Injury

In a rat liver transplantation model, hypoxia-induced HIF-1α expression protected mitochondrial function and Ca^2+^-ATPase activity to prevent I/R damage. As an important Ca^2+^ transporter, Ca^2+^-ATPase tightly controls intracellular Ca^2+^ levels. Ionized calcium is the ubiquitous second messenger that activates the signal cascade (Berridge, 1993; Brini and Carafoli, 2000; Berridge et al., 2003). Ca^2+^ signaling regulates a wide range of cellular and physiological processes, but prolonged elevation of intracellular free Ca^2+^ is toxic and can cause damage to cells (Orrenius et al., 2015). Therefore, secondary active transporters represented by plasma membrane Na^+^/Ca^2+^ exchangers will excrete excess Ca^2+^ in large quantities, thereby maintaining intracellular Ca^2+^ homeostasis. Decreased Ca^2+^-ATPase activity is an early manifestation of intestinal mucosal cells during ischemia–reperfusion injury. During intestinal ischemia, decreased Ca^2+^-ATPase activity can lead to intracellular calcium overload. During intestinal ischemia, the massive inactivation of Ca^2+^-ATPase allows the influx of extracellular calcium and greatly increases the concentration of calcium ions in the cytoplasm. Intracellular calcium-activated proteolytic enzyme produces a large number of free radicals. Oxygen free radicals destroy cell membrane lipids and can also lead to the inactivation of Na^+^-K^+^-ATP ATPase and Ca^2+^/Na^+^ exchange, which can promote Ca^2+^ influx and cause intracellular calcium overload (Lounsbury et al., 2000). At the same time, intracellular Ca^2+^ imbalance can lead to mitochondrial oxygen utilization disorder, which, in turn, leads to ATP synthesis disorder and cell damage. Hypoxic pretreatment (HP) can improve the hypoxia tolerance of small intestinal mucosal cells. Under hypoxia, HIF-1 increases Ca^2+^-ATPase activity and reduces apoptosis and pathological damage in small intestinal cells. HP may be an excellent way to promote the recovery of bowel function after transplantation (Ji et al., 2018).

### 2.4 The Role of Ion Transporters in the Pathogenesis of Liver Disease Under Hypoxic Conditions

#### 2.4.1 TRPC6 Can Induce the Expression of Hypoxia-Inducible Factor-1-α and Promote Multidrug Resistance of Hepatocellular Carcinoma Cells

TRPC6 is also a member of the transient receptor potential (TRP) channel superfamily and promotes the development of multidrug resistance in hepatocellular carcinoma (HCC). Hepatocellular carcinoma is a highly malignant tumor with low sensitivity to chemotherapy. Part of this is because it is prone to multidrug resistance (MDR). Among them, EMT, HIF1-α signaling, and DNA damage repair play important roles in the multidrug resistance of HCC (Zhang et al., 2007; Teicher, 2009; Van Zijl et al., 2009; Luo et al., 2014). Several studies have shown that MDR-related mechanisms such as EMT, hypoxia-induced HIF1-α signaling pathway, and DNA damage repair are closely related to intracellular calcium. Intracellular calcium is a multifunctional secondary messenger involved in many physiological and pathological processes. The calcium signaling pathway plays a vital role in tumor cells through apoptosis, proliferation, invasion, and metastasis. Intracellular calcium homeostasis is regulated by calcium channels/pumps. In oncology, changes in the expression of specific calcium channels and pumps are characteristic of certain cancers (Monteith et al., 2007). TRPC6 is expressed at low levels in normal hepatocytes, but is highly expressed in liver cancer samples. Studies have shown that the expression of TRPC6 is significantly increased in the hypoxic environment of liver cancer cells. The overexpression of TRPC6 causes a continuous increase in intracellular free calcium and regulates the EMT, HIF1-α signaling, and DNA damage repair mechanisms related to multidrug resistance to stimulate and enhance the resistance of liver cancer cells to multiple drugs. However, after pretreatment with the calcium chelator BAPTA-AM, TRPC6 interference was observed. Hepatocarcinoma cells show a significant decrease in drug resistance under stimulation by EMT, HIF1-α signaling, and DNA damage repair mechanisms. Mechanisms of EMT, HIF1-α signaling, and DNA damage repair have been reported to be regulated by upstream calcium-dependent protein phosphorylation, such as Erk, AKT, and STAT3 (Li and Melton, 2012; Liu et al., 2014; Pawlus et al., 2014; Xu et al., 2015). Studies have shown that calcium chelation reduces the expression of STAT3, suggesting that STAT3 activation acts as a downstream regulator in TRPC6/calcium signaling. Collectively, the TRPC6/calcium/STAT3 pathway can mediate mechanisms such as EMT, HIF1-α signaling, and DNA damage repair to promote multidrug resistance in HCC cells under hypoxia. Targeting TRPC6 in the treatment of liver cancer may be a new antitumor strategy, especially in combination with chemotherapy (Wen et al., 2016).

#### 2.4.2 Under Hypoxia, TRPC6 Stimulates Hepatic Stellate Cell Fibrosis

##### 2.4.2.1 During Liver Fibrosis, HIF-1α Promotes the Expression of TRPC6 Through the Notch Pathway

Hypoxia is a key factor regulating liver fibrosis, which is a self-healing and healing process for chronic liver damage and is closely related to the development of liver cirrhosis and hepatocellular carcinoma. However, it is currently known to be a dynamic process characterized by the excessive synthesis and deposition of the extracellular matrix (ECM), which destroys the normal structure of the liver and ultimately leads to organ dysfunction and failure (Bataller and Brenner, 2005). In the process of fibrosis, hepatic stellate cells (HSCs) are undoubtedly the main cells responsible for the excessive deposition of the extracellular matrix (ECM). Hypoxia acts as an environmental stress factor to activate oxygen-sensitive hepatic stellate cells, and stellate cell activation is a unique initiating factor of liver fibrosis (Shi et al., 2007). Stimulated by hypoxia, hepatic stellate cells undergo a complex activation process leading to increased ECM synthesis and deposition in fibrotic livers. Hypoxia can activate the transcription of hypoxia-inducible factor (HIF), and HIF-1α can induce the expression of key signaling components of the Notch pathway. The Notch pathway plays an extremely important role in the process of fibrosis (Kavian et al., 2012), inducing transcription factors to trigger the differentiation of fibroblasts into myofibroblasts (Gao et al., 2012). The key to the Notch pathway is the expression of the Notch intracellular domain (NICD) (Suwanjunee et al., 2008). Activated NICD can enter the nucleus to interact with transcriptional regulators, replace transcriptional co-repressors, recruit coactivators, and finally perform transcriptional activation of target genes (Lai, 2002). Qiang et al. (2012) showed that HIF1-α can promote the expression of Notch downstream genes by inducing the expression of NICD (Reya et al., 2001; Qiang et al., 2012). The transcriptional regulator NICD can activate the transcription of many ion channel genes, one of which is TRPC6 (Chigurupati et al., 2010).

##### 2.4.2.2 TRPC6 Can Promote ECM Deposition to Form Fibrosis

Under hypoxia, HIF-1α upregulates the expression of TRPC6 by inducing the expression of NICD. It is well known that Ca^2+^ channels are crucial in regulating growth control processes (Kunzelmann, 2005). Ca^2+^ influx mediated by TRPC6 channels directly activates calcineurin A expression. Increased calcineurin A activates various processes involved in myofibroblast and fibrotic transformation through its downstream activated T cell transcription effector nuclear factor (NFAT) (Kuwahara et al., 2006). Including ECM proteins such as α-SMA and collagen, which are activated by the transcriptional effector NFAT, both α-SMA and collagen mRNA transcript levels are significantly increased under hypoxia compared with normoxia. Furthermore, a previous study found that Smad3 is a main mediator of HSC fibrosis, especially in inducing collagen expression (Inagaki et al., 2001; Schnabl et al., 2001; Furukawa et al., 2003). Hypoxia-induced TRPC6 activates Smad2/3-dependent transforming growth factor-β signaling and promotes the upregulation of α smooth muscle actin, fibronectin, and collagen, which greatly promotes the transformation of activated hepatic stellate cells into myofibroblasts (Zhao et al., 2011). Taken together, hypoxia-induced TRPC6 activation leads to ECM protein deposition, which promotes the formation of liver fibrosis. TRPC6 appears to be a potential therapeutic target in the development of targeted intervention points for liver fibrotic diseases (Iyer et al., 2015).

#### 2.4.3 Effects of STIM on Hepatocellular Carcinoma Under Hypoxic Environment

##### 2.4.3.1 STIM Mediates SOCE Signaling Pathway

Matrix interacting molecules (STIMs) are a specialized class of single-channel transmembrane proteins ubiquitously expressed in the endoplasmic reticulum (ER) membrane. They typically associate with Orai ion channels in the plasma membrane (PM) to form calcium release-activated calcium (CRAC) channels. An intracellular signaling pathway called store-operated calcium entry (SOCE) is heavily dependent on CRAC channels. SOCE is one of the major pathways for calcium entry in non-excitable cells. The SOCE pathway is activated by ligand-induced depletion of ER calcium stores. STIM proteins acting as calcium sensors then sense this depletion and activate Orai ion channels through direct physical interactions to allow influx of calcium ions for storage refill and downstream signaling processes. SOCE regulates a variety of biological processes. Growing evidence suggests that SOCE even plays a key role in cancer cell proliferation, metastasis, and tumor neovascularization, as well as in antitumor immunity (Xie et al., 2016).

##### 2.4.3.2 In Hypoxic Environment, STIM Interacts With HIF-1a to Promote Hepatocarcinogenesis

Hypoxia and intracellular Ca^2+^ transients are the basic characteristics of tumors, signaling cascades initiated or regulated by HIF-1 are critical for the process of tumorigenesis (Neumann et al., 2005), and STIM1 mediates SOCE activation and promotes tumor invasion and migration (Van de Vijver et al., 2002; Tsai et al., 2014). However, there is no clear consensus on the relationship between HIF-1 and STIM1. We were surprised to find that some studies have shown that in the hypoxic environment of liver cancer cells, hypoxia-induced HIF-1a promotes STIM1 expression and SOCE in liver cancer cells by directly binding to the STIM1 promoter. SOCE can positively regulate the expression of Vascular endothelial growth factor (VEGF) and other growth factors to promote the proliferation and migration of hepatoma cells. Meanwhile, SOCE stabilizes HIF-1a by activating CaMKII and p300, thereby preventing HIF-1a degradation. This regulatory loop aggravates the hypoxic microenvironment and accelerates the growth of tumors. Giving the HIF-1 inhibitor YC-1 or knocking out HIF1a can significantly reduce hypoxia-enhanced STIM1 and inhibit the occurrence of tumors. These results suggest that STIM1 and HIF-1 are interdependent and regulated in controlling Ca^2+^ mobilization and hypoxic tumor growth and may become potential targets for early cancer intervention (Y. Li et al., 2015).

#### 2.4.4 STIM Mediates Hepatic Ischemia-Reperfusion (I/R) Injury Under Hypoxic Conditions

Hepatic ischemia–reperfusion (I/R) injury is unavoidable during trauma, elective liver resection, shock, or liver transplantation and has adverse effects on patients’ health (Inoue et al., 2014; Li et al., 2014). Hepatic I/R injury is a complex and multifactorial pathophysiological process involving the effects of inflammatory cytokines, ROS, and apoptosis (Sun et al., 2014). Furthermore, ROS induces the activation of Kupffer cells, which, in turn, produce a large amount of inflammatory cytokines and oxygen free radicals, further aggravating liver damage (Kalogeris et al., 2014; Tao et al., 2014). Under hypoxic conditions, the levels of the STIM1 gene and protein in Kupffer cells are significantly upregulated. The STIM protein is a calcium storage sensor that mediates cell responses to various stress conditions, including elevated ROS and hypoxia (Zhou et al., 2010). STIM can release and enter the cell to cause continuous Ca^2+^ overload and promote the production of the transcription factor NF-κB. NF-κB plays an important role in mediating inflammation by promoting the release of proinflammatory cytokines (Haddad, 2002). In I/R injury, the activation of NF-κB is related to an increase in tumor necrosis factor-α, interleukin-1β, and interleukin-6 (Lan et al., 2013). STIM1 gene knockout can reduce inflammation, oxidative stress, and apoptosis in cells exposed to hypoxia. Therefore, we believe that STIM1 can be used as a potential therapeutic target to improve I/R injury (Y. Li et al., 2018).

### 2.5 Ion Transporters Affect the Occurrence of Pancreatic Diseases Under Hypoxic Conditions

#### 2.5.1 STIM1 Promotes the Invasion and Metastasis of Pancreatic Ductal Carcinoma Under Hypoxia

Pancreatic ductal adenocarcinoma (PDAC) is among the most aggressive and refractory cancers. Numerous studies have shown that the growth, invasion, and metastasis of PDAC are related to calcium ions and calcium channels (Morrone et al., 2016; Shapovalov et al., 2016; Yeung et al., 2017). Hypoxia is a common microenvironmental feature in tumors that promotes tumor growth and metastasis, especially in PDAC. Substantial evidence shows that the expression of hypoxia-inducible factor-1α in pancreatic cancer tissue is positively correlated with the expression of STIM1. HIF-1α can bind the HER2-3 region of the STIM1 promoter to regulate the transcription of STIM1. When shRNA was used to knock out hypoxia-inducible factor-1α in pancreatic cancer cells, STIM1 mRNA and protein were significantly reduced in the pancreatic cancer cells with HIF-1α gene knockout. HIF-1 frequently upregulates the expression of STIM1 in pancreatic cancer cells. STIM1 can upregulate vimentin and decrease the expression of E-cadherin, indicating that STIM1 may play a key role in PDAC oncogenic transformation, cell growth and invasion, and epithelial-mesenchymal transition (EMT). Moreover, studies have shown that high expression of STIM1 is significantly related to the tumor grade and early recurrence, which are important clinical factors affecting the prognosis of PDAC patients. Therefore, detecting the expression level of STIM1 in PDAC tissues can be used as a new method to predict the prognosis of PDAC patients. The HIF1α/STIM1 axis may be a potential therapeutic target for PDAC (Wang et al., 2019).

#### 2.5.2 TRPC6 Promotes Pancreatic Stellate Cell Fibrosis Under Hypoxia

Pancreatic cancer is characterized by massive fibrosis mainly caused by activated pancreatic stellate cells (PSCs). Pancreatic stellate cells are the predominant mesenchymal cell type in the PDAC stroma and are responsible for the overproduction of extracellular matrix proteins. Pancreatic stellate cells are primarily affected by transforming growth factor beta (TGF-β), tumor necrosis factor alpha (TNF-α), and other factors such as platelet-derived growth factor (PDGF) or interleukin-8 (IL- 8) Activate. In the hypoxic environment of PDAC, hypoxia can promote the secretion of matrix proteins and growth factors to activate pancreatic stellate cells, but the specific mechanism of the action is not fully understood in molecular details. In recent years, studies have found that TRPC6 and Ca^2+^ signaling are involved in the activation of pancreatic stellate cells. Further studies have shown that most growth factors and chemokines trigger their actions through G protein-coupled receptors (GPCRs). The TRPC6 channel is the effector protein of these G protein-coupled receptor pathways. In the hypoxic tumor microenvironment, the TRPC6 channel participates in the continuous activation of PSCs, which is a typical feature of the PDAC microenvironment. At the same time, it provides a potential target for the treatment of pancreatic ductal carcinoma (Nielsen et al., 2017).

### 2.6 Inhibition of NCX Expression Prevents Hypoxia-Induced Pancreatic β-cell Damage

The Na^+^/Ca^2+^ exchanger (NCX) belongs to the antiporter family and is a major Ca^2+^ regulatory protein, expressed in all excitable and many non-excitable cells, and transports Ca^2+^ across the plasma membrane (Khananshvili, 2021) (Quednau et al., 2004). Islet B cells express NCX (Van Eylen et al., 1997). It is not only involved in maintaining glucose homeostasis by regulating Ca^2+^-dependent insulin secretion (Hamming et al., 2010), but also in cell survival or death through its anti-apoptotic or pro-apoptotic effects, respectively (Diaz-Horta et al., 2002; Nguidjoe et al., 2011). Immediately exposed to low oxygen tension, the transplanted islets release a large amount of HMGB1, which triggers innate immune rejection and activates DCs, NKT cells, and neutrophils to produce IFN-γ, and ultimately pancreatic transplantation fails. Therefore, the release of HMGB1 plays a crucial role in this process. Experiments have demonstrated that the release of HMGB1 from transplanted islets is due to hypoxia-induced Ca^2+^ influx into β cells through Na^+^/Ca^2+^ exchanger (NCX), and Ca^2+^ stimulates islet β cells to induce the release of HMGB1. Furthermore, hypoxia-induced β-cell damage is prevented by pretreatment with NCX-specific inhibitors prior to transplantation, resulting in protection and long-term survival of transplanted islets (Mera et al., 2013).

## 3 Conclusion

In summary, under hypoxia, ion-transporting proteins mediate various signaling pathways in various organs of the digestive system (Table 1), thereby regulating cell functions and participating in the pathophysiological process of the development of various diseases of the digestive system. HIF and ion-transporting proteins have become a new research hotspot and may become new markers for the diagnosis, treatment, and prognosis assessment of diseases of the digestive system. The development of drugs related to ion-transporting proteins and hypoxia could also become a new direction for the treatment of digestive system diseases.

**TABLE 1:** The effects of ion-transporting proteins on the digestive system under hypoxic conditions.

Digestive system	Ion-transporting proteins	The role of ion-transporting proteins in the digestive system under hypoxia	Mechanism	References
Esophagus	V-ATPase	Energy metabolism of esophageal cancer	V-ATPase can promote the expression of mammalian target of rapamycin (MTOR). MTOR increases glucose uptake and expression of glucose transporter 1 (GLUT1). In addition, experiments showed that V-ATPase can also promote HIF-1 and other glycolytic genes such as HK2, phosphofructokinase-1 (PFK1), enolase 1 (ENO1), PKM2, LDHA and pyruvate dehydrogenase thiamine kinase isoenzyme (PDK1) expression	Son et al. (2016)
Stomach	V-ATPase	Promote multidrug resistance in gastric cancer cells	V-ATPase increases HIF-1α translation to promote P-gp and MRP1 expression, thereby reducing intracellular drug accumulation and enhancing the drug resistance of esophageal cancer cells	Chen et al. (2012)
	MCU	MCU can promote the invasion of gastric cancer	MCU can increase the level of MMP, destroy the balance of ECM, reduce E-cadherin and promote EMT, enhance the transcription of HIF-1α and promote the expression of VEGF to induce the proliferation, migration and invasion of gastric cancer cells	Wang et al. (2020)
Large intestine	K_2P_5.1	Involved in the pathogenesis of inflammatory bowel disease	HIF-1 can clone the promoter of K2P5.1, and the dysregulation of K2P5.1 expression is related to the pathogenesis of immune and inflammatory diseases	Endo et al. (2019)
	Piezo1 and MCU	Piezo1 and MCU are involved in colon cancer metastasis in hypoxic environment	Piezo1 and MCU are likely to play a role in colon cancer cell metastasis through the Piezo1-MCU-HIF-1α-VEGF pathway	Sun et al. (2020)
	BKca	Related to the electrophysiology of mesenteric artery vascular smooth muscle cells (VSMCs)	Acute hypoxia activates the BKca channel on the mesenteric artery smooth muscle cell membrane to cause K^+^ efflux, and the vascular smooth muscle cell hyperpolarizes and causes vasodilation, thereby improving the mesenteric microcirculation	Ma et al. (2011)
	IKCa	Increase the permeability of mesenteric cells	Metabolic stress secondary to chemical hypoxia causes a rapid increase in the activity of IKCa channels in the basolateral membrane of natural human intestinal epithelial cells and a significant increase in the whole cell conductance on the basal side of human colonic crypt. Furthermore, the paracellular permeability (GS) of the colonic membrane is doubled, and the increase in mucosal permeability may lead to bacterial translocation, sepsis and multiple organ failure	Loganathan et al. (2011)
	TrpC5	Tumor metastasis in colon cancer patients may be related to transient receptor potential channel 5 (TrpC5)	TrpC5 can induce colon cancer cell epithelial-mesenchymal transition through the HIF-1α-Twist signaling pathway, thereby promoting tumor cell metastasis	Chen et al. (2017)
Small intestine	Ca^2+^-ATPase	Hypoxia-induced HIF-1α expression can protect mitochondrial function and Ca^2+^-ATPase activity to prevent I/R damage	Under hypoxia, HIF-1 increases the activity of Ca^2+^-ATPase, thereby avoiding calcium overload and reducing apoptosis and pathological damage in small intestinal cells	Ji et al. (2018)
Liver	TRPC6	The HIF1-α signaling pathway can stimulate liver cancer cells to develop multidrug resistance by affecting the expression of TRPC6	Under hypoxia stimulation, TRPC6 mRNA transcription significantly increases. The overexpression of TRPC6 causes the continuous increase of intracellular free calcium and regulates the multidrug resistance-related EMT, HIF1-α signaling and DNA damage repair mechanisms to stimulate and enhance the resistance of liver cancer cells to multiple drugs	Wen et al. (2016)
	TRPC6	HIF1α can affect liver fibrosis by regulating the expression of TRPC6	HIF1α stimulates the expression of TRPC6 to change the intracellular Ca^2+^ concentration, thereby activating the expression of calmodulin A. Calmodulin A regulates the NFAT factor and activates various processes in the process of myofibroblast and fibrosis transformation. Furthermore, hypoxia-induced TRPC6 activates Smad2/3-dependent transforming growth factor-β signaling and promotes the upregulation of smooth muscle actin, fibronectin, and collagen, thereby promoting myofibroblasts in activated hepatic stellate cell formation	Kuwahara et al. (2006); Zhao et al. (2011)
	STIM1	STIM1 mediates the invasion and migration of liver cancer cells under the stimulation of HIF1-a	Hypoxia-induced HIF-1 directly binds the STIM1 promoter to promote STIM1 expression and SOCE in liver cancer cells. SOCE stabilizes HIF-1a by activating CaMKII and p300. This regulatory loop intensifies the hypoxic microenvironment and accelerates the growth of tumors	Li et al. (2015)
	STIM1	STIM1 is related to I/R damage	Under hypoxia, the STIM1 gene and protein levels in Kupffer cells are significantly upregulated. The overexpression of STIM can increase ROS, and ROS induces Kupffer cell activation, which, in turn, produces numerous inflammatory cytokines and oxygen free radicals and aggravates I/R damage	Li et al. (2018)
Pancreas	STIM1	HIF-1α stimulates the expression of STIM1, which, in turn, participates in the growth and metastasis of pancreatic cancer cells。	HIF-1α can bind the HER2-3 region of the STIM1 promoter to regulate the transcription of STIM1. STIM1 can upregulate vimentin and decrease the expression of E-cadherin, thereby promoting the proliferation and invasion of pancreatic cancer cells	Wang et al. (2019)
	TRPC6	TRPC6 under hypoxia is related to pancreatic cancer fibrosis	In the hypoxic tumor microenvironment, TRPC6 can continuously activate pancreatic stellate cells in pancreatic cancer cells, which, in turn, promotes a large amount of fibrosis	Nielsen et al. (2017)
	NCX	NCX is related to the immune response of pancreas transplantation	Hypoxia can activate NCX and increase Ca^2+^ in β cells, which, in turn, stimulates the release of HMGB1 from islet β cells. HMGB1 mediates immune rejection	Mera et al. (2013)
